# Low-Computational-Cost Technique for Modeling Macro Fiber Composite Piezoelectric Actuators Using Finite Element Method

**DOI:** 10.3390/ma14154316

**Published:** 2021-08-02

**Authors:** Diaa Emad, Mohamed A. Fanni, Abdelfatah M. Mohamed, Shigeo Yoshida

**Affiliations:** 1 Mechatronics and Robotics Engineering Department, Egypt-Japan University of Science and Technology (E-JUST), New Borg El-Arab, 21934 Alexandria, Egypt; mohamed.fanni@ejust.edu.eg (M.A.F.); abdelfatah.mohamed@ejust.edu.eg (A.M.M.); 2 Mechatronics Department, Faculty of Engineering, Ain Shams University, 11517 Cairo, Egypt; 3 Production Engineering and Mechanical Design Department, Faculty of Engineering, Mansoura University, 35516 Mansoura, Egypt; 4 Electrical Engineering Department, Faculty of Engineering, Assiut University, 71518 Assiut, Egypt; 5 Research Institute for Applied Mechanics, Kyushu University, Fukuoka 816-8580, Japan; yoshidas@riam.kyushu-u.ac.jp; 6 Institute of Ocean Energy, Saga University, Saga 840-8502, Japan

**Keywords:** piezoelectric actuator, macro fiber composite, morphing wing, finite element analysis, smart structure

## Abstract

The large number of interdigitated electrodes (IDEs) in a macro fiber composite (MFC) piezoelectric actuator dictates using a very fine finite element (FE) mesh that requires extremely large computational costs, especially with a large number of actuators. The situation becomes infeasible if repeated finite element simulations are required, as in control tasks. In this paper, an efficient technique is proposed for modeling MFC using a finite element method. The proposed technique replaces the MFC actuator with an equivalent simple monolithic piezoceramic actuator using two electrodes only, which dramatically reduces the computational costs. The proposed technique was proven theoretically since it generates the same electric field, strain, and displacement as the physical MFC. Then, it was validated with the detailed FE model using the actual number of IDEs, as well as with experimental tests using triaxial rosette strain gauges. The computational costs for the simplified model compared with the detailed model were dramatically reduced by about 74% for memory usage, 99% for result file size, and 98.6% for computational time. Furthermore, the experimental results successfully verified the proposed technique with good consistency. To show the effectiveness of the proposed technique, it was used to simulate a morphing wing covered almost entirely by MFCs with low computational cost.

## 1. Introduction

A smart structure is a well-known research topic in the mechanical engineering field, which has a long history of challenges and aspirations. Recently, aeronautic applications, especially small aircraft with morphing wings, have employed piezoelectric actuators for improving aerodynamic characteristics. Some of the required aerodynamic improvements are to reduce fuel consumption, reduce drag, and enhance flight conditions for navigation and maneuvering. The conventional piezoelectric materials have some limitations such as their very weak behavior at high mechanical stress, which leads to them cracking easily during the bonding process and operation. Moreover, its formation performance on curved surfaces is weak [1]. Therefore, a piezo-composite material is proposed to solve the challenges and problems encountered with conventional piezo ceramic material.

The piezo-composite actuator is composed of active piezoceramic fibers embedded in a polyimide film. The piezoceramic crystal gains high strength in its fibrous form, which protects the piezo fibers from cracking, and the polymer matrix allows high flexibility for the device to be easily shaped with curved surfaces [2].

The macro fiber composite (MFC) piezoelectric material is widely used in smart structure applications. This MFC is composed of rectangular rods of piezoceramic material (PZT-5A) inserted between Kapton layers and interdigitated electrodes (IDEs) [3,4]. The MFC retains the merits of the previous active fiber composite (AFC), as it produces large longitudinal deformation by using reasonable voltage owing to the large value of the longitudinal piezoelectric constant, d_33_. MFC features uniform and repeatable fabrication [5]. A full description of the fabrication process and properties of MFC can be found in [2,6,7,8].

Today, Smart Materials Corp. produces MFC in two operational modes: d_33_ and d_31_. The first mode is based on the d_33_ effect and is called an elongator, which is used as a powerful actuator and sensitive sensor. Both electric field and induced strain are generated in the piezo-fiber direction (d_33_ direction). This mode works in a voltage range from −500 V to +1500 V, where it produces contraction deformation until −500 V and elongation deformation up to +1500 V, explaining its name (elongator) because it generates larger elongation strain compared to contraction strain. The second mode is based on the d_31_ principle, which is used as a low-impendence sensor and energy generator. Its voltage range is from −60 V to +360 V and is called contractor as it produces more contraction strain than elongation strain for this voltage range [4]. Here, in this work, we focus mainly on the MFC-F1 type with 45° fiber orientation operated in d_33_ mode as a powerful actuator.

Finite element analysis is a powerful numerical technique to model smart structures equipped with piezoelectric materials. Du et al. [9] used the finite element method to model and evaluate piezoelectric unit distributions for piezoelectric energy-harvesting applications. Finite element simulations were used to identify the properties of a piezoelectric element using the neural network method in [10]. In addition, a neural network model was introduced in [11] to investigate the piezoelectric effect on the natural frequency of a plate using the finite element method. Another numerical method using a representative volume element (RVE) was presented by Medeiros et al. [12] for evaluating the effective properties of smart piezoelectric composite materials. Furthermore, finite element analysis was used in [13] to model a cantilever beam embedded with piezoelectric plates. This study focused on finding the optimal distribution of the piezoelectric plates to control the torsional vibrations. Several finite element simulations were conducted in [14] to deduce the piezoelectric coefficient of piezoelectric particulate composite for energy harvesting application.

Computational costs and simulation speeds are still crucial issues today in finite element analysis. In the research field, several techniques have been developed to reduce computational costs and enhance simulation speeds. Zhang [15] developed a novel algorithm for total Lagrangian explicit dynamics (TLED) FE using a direct Jacobian formulation. The components of strain and stress were implicitly formulated in the nodal forces using the Jacobian operator. This technique showed speed enhancements of the GPU, as well as low CPU consumption for hyper-elastic FE models. Another technique was presented in [16] to improve computational efficiency. This article introduced a new computation approach for the smoothed particle FE method in GPU parallel computing. The reduced-order modeling method is widely used in FE analysis to solve the complexity of multiscale FE models. One of the implementations of such an approach was discussed by the authors in [17,18]. The authors implemented the reduced model approach to solve the complexity of the finite element square method, which was used to describe the nonlinearities of material microstructures. However, the FE square method is impracticable due to its huge computational costs. Hence, the authors used the high-performance reduced FE square method to overcome the huge computational effort and evaluated their work in [19] for an industrial multiscale model. Many research works utilize the reduced-order model (ROM) technique to enhance the computational costs in different FE applications, some of which can be found in the literature [20,21,22,23].

Wang et al. [24,25] used an MFC piezoelectric actuator for active shape control of the morphing wing. The interaction among structural dynamics, unsteady aerodynamics, and MFC actuation was investigated to achieve active dynamic morphing for flexible wings. A bimorph configuration of MFCs on a flexible cantilever beam was modeled with 45° fiber orientation to obtain both bending and torsional deformations for active shape control. The detailed modeling of the MFC using FEM was not presented in these works, which leaves open questions without answers.

Bowen et al. [26,27] presented the MFC characteristics by creating matrices for compliance, relative permittivity, and piezoelectric constants. They used ANSYS software to develop an FE model of the MFC piezoelectric actuator bonded to a cantilever beam. This FE model of MFC has equal voltage planes at 5 mm spacing through the longitudinal axis of MFC to ensure a constant electric field aligned with the longitudinal axis of MFC. The finite element model was validated successfully by experimental measurements on a cantilever beam. A confusing issue in this work was the usage of 5 mm as the spacing between the electrodes, whereas its actual value is 0.5 mm according to many references [28,29,30,31,32] and the MFC data sheet [4].

Huang [33] indicated the problem arising from modeling the MFC elements with opposite pooling directions between adjacent pairs of electrodes. According to [33], this problem results from the significant strain break that exists at the junction of two electrodes. Consequently, the author proposed the usage of piezoelectric elements with the same direction of polarization, which resulted in a high voltage difference between the first and last electrodes. The author carried out numerical tests to calculate free strain and blocking force, as well as experimental tests to validate his proposed technique. Although the author’s claim of solving the strain break problem was associated with modeling piezoelectric elements of opposite polarization directions, he was forced to use a very fine net due to the small electrode spacing (0.5 mm), which resulted in a large problem size and large computational time. Such a fine net hinders the FE modeling of many MFCs employed in large flexible structures.

Another work for designing and testing micro aerial vehicles using morphing wings controlled by MCF piezoelectric actuators was presented by Kochersberger et al. [34]. The authors stated that modeling MFC in simulating a morphing wing is challenging. Therefore, the authors followed a technique developed by Gustafson [35], which assumes that the piezoelectric expansion coefficient is equivalent to the thermal expansion coefficient. This thermal expansion coefficient was calculated from the piezoelectric constants of MFC.

From the above-discussed previous works, one can find that some confusing issues and challenges have hindered progress in MFC modeling. Thus, our aim in this work was to propose a new technique for efficient modeling of MFC actuator using the FE method which requires low computational cost and memory consumption. This enables the accurate and efficient modeling of a large number of MFCs employed in smart structures. The main contribution of this paper is the realization that the restrictions dictated in practice should not also be restrictions in modeling. A preliminary idea was presented in [36] for the MFC–P1 type.

The article is organized as follows: after introducing piezoelectric composites and MFC, as well as reviewing the previous research works in this field in Section 1, Section 2 describes the mechanical and electrical properties of homogenized MFC. Section 3 presents theoretically the newly proposed technique for modeling MFC. Section 4 shows the simulation validations with its results, while Section 5 presents the experimental verification of the proposed technique of modeling MFC. Then, the experimental results are discussed in Section 6. In Section 7, the newly proposed technique for modeling MFC is applied to simulate a morphing wing covered with multiple MFCs. Lastly, Section 8 presents the conclusion of the presented work.

## 2. Mechanical and Electric Properties of MFC

### 2.1. MFC Homogenization Model

Piezoelectric material behaves linearly for low electric fields and small mechanical stresses, while it has some nonlinearity for high electric fields and large mechanical stresses [37]. The linear electromechanical equations for the piezoelectric materials are as follows:

(1) $$\varepsilon_{i}={\;S}_{\mathrm{ij}}^{E}{\;\sigma }_{i}+d_{\mathrm{mi}}E_{m},$$

(2) $$D_{m}={\;d}_{\mathrm{mi}}{\;\sigma }_{i}+\xi_{\mathrm{ik}}^{\sigma }{\;E}_{k},$$

where i, j = 1,2, …, 6, m, k = 1, 2, 3 referring to the material (*x*, *y*, *z*) coordinate system respectively, $\sigma_{i}$ is a stress vector (N/m^2^), $\varepsilon_{i}$ is a strain vector (m/m), $E_{m}$ is a vector of the applied electric field (V/m), $\xi_{\mathrm{ik}}^{\sigma }$ is the permittivity at constant stress (F/m), $d_{\mathrm{mi}}$ is a matrix of piezoelectric strain constant (m/V), $S_{\mathrm{ij}}^{E}$ is a matrix of compliance coefficients at a constant electric field (m^2^/N), and $D_{m}$ is a vector of electric displacement (C/m^2^).

The first equation represents the inverse piezoelectric effect as an actuator, while the second equation represents the direct piezoelectric effect as a sensor. Here, we focus on the piezoelectric actuator. The homogenized compliance matrix for the piezo-composite actuator is considered a transversely isotropic material which is a special form of orthotropic material [38]. Equation (3) represents the compliance matrix of an orthotropic material in ANSYS format, where the *z*-direction is aligned with the piezo-fiber direction.

(3) $$S=\left[\begin{array}{cccccc} \frac{1}{E_{1}} & \frac{-\nu_{21}}{E_{2}} & \frac{-\nu_{31}}{E_{3}} & 0 & 0 & 0\\ \frac{-\nu_{12}}{E_{1}} & \frac{1}{E_{2}} & \frac{-\nu_{32}}{E_{3}} & 0 & 0 & 0\\ \frac{-\nu_{13}}{E_{1}} & \frac{-\nu_{23}}{E_{2}} & \frac{1}{E_{3}} & 0 & 0 & 0\\ 0 & 0 & 0 & \frac{1}{G_{12}} & 0 & 0\\ 0 & 0 & 0 & 0 & \frac{1}{G_{23}} & 0\\ 0 & 0 & 0 & 0 & 0 & \frac{1}{G_{13}} \end{array}\right]$$

where E is Young’s modulus, υ is Poisson’s ratio, and G is the shear modulus of the homogenized MFC material. The piezoelectric strain constant $(d_{\mathrm{mi}}$) can be represented in ANSYS format as

(4) $$d=\left[\begin{array}{ccc} 0 & 0 & d_{31}\\ 0 & 0 & d_{32}\\ 0 & 0 & d_{33}\\ 0 & 0 & 0\\ 0 & d_{24} & 0\\ d_{15} & 0 & 0 \end{array}\right]$$

The parameters for the homogenized matrix used in the finite element model in this work are according to Williams’s experimental measurements for MFC [29], which match with the manufacturer’s datasheet [4]. A comparison between Williams’s experimental measurements and datasheet values is shown in Table 1.

### 2.2. Nonlinearity Effect and Free Strain

**Figure 1:**
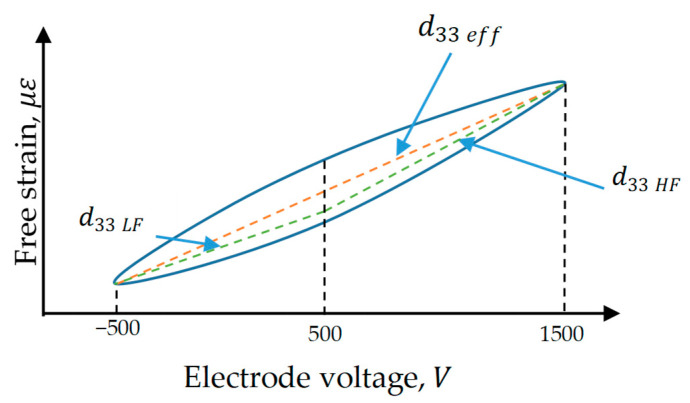
Longitudinal free strain versus applied electrode voltage showing the piezoelectric constant variation with the voltage range.

The piezoelectric constants $d_{\mathrm{mi}}$ are important parameters in developing a finite element model of MFC. They are equal to the amount of free strain induced by a unit applied electric field without external loads. The peak-to-peak free strain is defined as the net induced strain from the piezoelectric material without any external loads and from the peak-to-peak electrode voltage, as shown in Figure 1. Any physical piezoelectric actuator has nonlinear behavior, which is notable in MFC performance for its composite structure and its interdigitated electrode scheme [3,39,40]. Hence, some investigations are needed to determine the effective values of piezoelectric constants suitable for finite element analysis of MFC.

Let us simply consider the nonlinear constitutive equation for the actuator piezoelectric continuum as

(5) $$\varepsilon_{T}={\;\varepsilon }_{M}+\varepsilon_{E}+{\;\varepsilon }_{n}$$

where,${\;\varepsilon }_{T}$ is the total strain tensor, $\varepsilon_{M}$ is the strain due to the mechanical stress tensor ($\varepsilon_{M}=S_{\mathrm{ij}}^{E}{\;\sigma }_{i}$), $\varepsilon_{E}$ is the strain resulting from the applied electric field ($\varepsilon_{E}={\;d}_{\mathrm{mi}}E_{m}$), and $\varepsilon_{n}$ is the equivalent nonlinear strain for higher-order terms [40].

Considering free strain (zero mechanical stress), Equation (5) can be reduced to

(6) $$\varepsilon_{f}=\varepsilon_{E}+{\;\varepsilon }_{n}$$

where, $\varepsilon_{f\;}$ is the free strain induced by MFC due to applying an electric field between its interdigitated electrodes.

MFC piezo-composite actuators, like any other piezoceramic materials, have intrinsic nonlinear behavior due to hysteresis and creep effects. It is indicated experimentally in Table 2 [40] that the nonlinear effects in MFC decrease as the voltage range increases at zero mechanical strain (free strain). As an example, it is stated in this table that the nonlinear ($d_{33}$) is 396 pm/V with a standard deviation of 69.4 pm/V at zero offset voltage. On the other hand, at 500 V offset, the nonlinear ($d_{33}$) is 56.8 pm/V with a standard deviation of 10.5 pm/V. Moreover, it is indicated in Tables 3–5 in [40] that the MFC behaves more linearly upon increasing the external load stress.

In addition, Figure 9 in the work published in [41] showed experimentally that the changes in ($d_{33}$) due to nonlinear effects decreased as voltage range increased. It was declared in this work that MFC’s nonlinear performance in the negative voltage range differs from its nonlinear performance in the positive voltage range. Additionally, it was stated in this work that a single MFC actuator has nearly the same nonlinear behavior as a composite MFC bonded to an aluminum sheet. Therefore, the effects of other mechanical factors on the nonlinear behavior of MFC can be neglected in the case of bonding MFC to a substrate.

Many research works handled the undesired nonlinear characteristics by adding a feedforward compensator to the control strategy to compensate for these nonlinearities [42,43]. The feedforward compensator can be designed using different hysteresis models, which can be found in the literature [44,45,46,47]. The dynamic process of MFC can be represented as a linear model in the control system with a feedforward compensator proposed in the control loop. Therefore, Equation (6) can be approximated by a piecewise linear model to get the effective piezoelectric constants, as shown in Table 2, which were obtained from the manufacturer datasheet [4]. This table supplies users with two values for piezoelectric constants according to the working range of the electric field. For a high electric field larger than 1 kV/mm, the longitudinal piezoelectric constant ($d_{33}$) is 460 pm/V (picometer per volt) and the transverse piezoelectric constant ($d_{31}$) is −210 pm/V. For a low electric field of less than 1 kV/m, ($d_{33}$) is 400 pm/V and ($d_{31}$) is −170 pm/V. The positive and negative signs in the piezoelectric constants represent extension and contraction, respectively. Furthermore, the approximate free strain induced by applying an electric voltage for the high piezoelectric constant ($d_{33}$) is about 0.9 ppm/V (parts per million per volt) for a high electric field and about 0.75 ppm/V for a low electric field.

In a conclusion, the linearity assumption in this study is valid for the high electric field as the nonlinear effects decrease in the high voltage range. On the other hand, in the low electric field, the linearity assumption can also be valid if we add a feedforward compensator in the control loop or use a robust controller for accurate positioning application.

The average electric field is defined as the applied voltage between two consecutive electrodes divided by the center-to-center electrode space. In MFC, the spacing between two consecutive electrodes (electrode pitch) is 0.5 mm. By neglecting the electrode width (~85 µm), the center-to-center electrode space becomes equal to the electrode pitch and, hence, the electric field of 1 kV/m corresponds to 500 V. Therefore, the high electric field can be taken from 500 to 1500 V and the low electric field can be taken from −500 to 500 V, as shown in Figure 1.

The red dashed line in Figure 1 represents the linear model of MFC free strain, where its inclination is the effective piezoelectric constant ($d_{33\;\mathrm{eff}}$) used in the past datasheet published by the manufacturer. The green lines represent the piecewise linear model with two piezoelectric constants, $d_{33\;\mathrm{HF}}$ and $d_{33\;\mathrm{LF}}$, for the high and low electric fields, respectively. The values of these constants are given in Table 2.

## 3. Methodology of the Proposed Technique for Modeling MFC

### 3.1. Description of the FE Model of MFC

**Figure 2:**
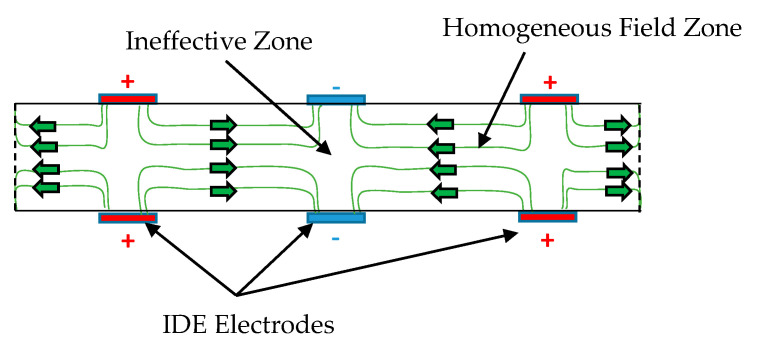
Sketch for the structure of homogenized MFC with interdigitated electrodes.

**Figure 3:**
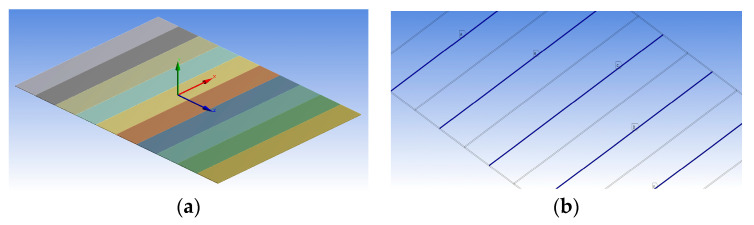
(**a**) FEM of multiple segments of piezoelectric actuators of MFC with the coordinate system. (**b**) Blue surfaces indicate positive voltage and gray surfaces indicate zero voltage in the finite element model.

The homogenized MFC is considered a monolithic piezoelectric actuator with interdigitated electrodes on the upper and lower surfaces, as shown in the simplified sketch of Figure 2. The equivalent finite element model of MFC is displayed in Figure 3.

The MFC is represented in the FE model with segments of piezoelectric material in the longitudinal *z*-direction linked together with faces of constant voltages that represent the interdigitated electrodes.

It is to be noted that the pooling direction changes between each successive segment. Moreover, the voltage changes its polarity along with the IDEs; hence, the electric field changes its direction between each successive segment, as illustrated in Figure 2. As a result, the electric field directions in all segments are the same as or opposite to the pooling directions and, therefore, all segments are subjected to either elongation or contraction, respectively.

### 3.2. Hypothesis of the New Technique for Modeling MFC

The homogenized model of MFC with its IDEs is represented as several segments of piezo-ceramic material connected along its longitudinal direction. Each segment has a longitudinal length of 0.5 mm. A constant voltage difference is employed for each segment between its extreme electrodes. Let us assume that the IDEs have negligible width, and that each segment produces a uniform electric field; then, the electric field in the longitudinal direction can be expressed as

(7) $$E_{3}=\frac{V}{t},$$

where $E_{3}$ is the electric field produced at each segment, V is the voltage difference employed between the extreme electrodes for each segment, and t is the longitudinal length of each segment (0.5 mm).

Hence, the free strain for each piezoelectric segment is the same in magnitude and direction and is calculated as

(8) $$\varepsilon_{3\;\mathrm{free}}=d_{33\;}\times E_{3}.$$

Our presented study is established on the basis of our understanding of the motives behind the ability of MFC to generate large deformation in contrast to the conventional monolithic piezoelectric material. A huge deformation can be produced from a piezoelectric actuator if the strain generated in the conventional through-thickness direction (lateral axis) can be generated across the longitudinal direction. Hence, to generate the same amount of strain through the longitudinal axis, the electric field should be employed through the longitudinal axis instead of the usual method for electric field employed over its thickness, to utilize the large $d_{33}$. However, to employ this electric field through the longitudinal axis of the piezo material equivalent to that employed in the piezo thickness, a huge voltage difference is required between the electrodes. Practically, there is a constraint on the maximum voltage that can be applied, say 1500 V. Therefore, researchers created MFC with a large number of IDEs distributed over the longitudinal direction with a small pitch of 0.5 mm. Every two consecutive electrodes are responsible for supplying the required voltage to the piezoelectric segment located between them. Every two adjacent piezo segments have opposite directions of polarization, as shown in Figure 2. This design permits applying a maximum voltage between every two successive electrodes, consequently obtaining large strain based on Equations (7) and (8) regarding the small spacing, t. Thanks to the high strains generated in numerous piezoelectric segments, the total deformation of MFC is dramatically increased compared with the conventional monolithic piezo-ceramic material.

From our point of view, this is equivalent to applying a huge voltage between the longitudinal extreme electrodes of a monolithic piezo-ceramic material with a single pooling direction. The huge new voltage difference, $V_{\mathrm{new}}$, can be calculated from the applied voltage on the IDEs of the physical MFC, V, and the number of piezoelectric segments, N, as illustrated in Equation (9). In addition, Equation (10) declares the dependency of N on the active length of MFC actuator, $L_{\mathrm{active}}$.

(9) $$V_{\mathrm{new}}=N\times V$$

(10) $$N=\frac{L_{\mathrm{active}}}{t}$$

The similarity between the newly proposed method with a single direction of polarization and the physical configuration with an opposite direction of polarization is proven by remarking that both techniques generate an equal electrical field, equal free strain, and equal deformation, as illustrated below.

Using Equations (7), (9) and (10), the electric field generated by the proposed method is revealed as

(11) $$E_{3\;\mathrm{new}}=\frac{V_{\mathrm{new}}}{L_{\mathrm{active}}}=\frac{N\times V}{N\times t}=\frac{V}{t}=E_{3}$$

Equations (8) and (11) reveal that both approaches generate equivalent free strain as they have the same electric field. Since equivalent free strain is applied on equivalent longitudinal lengths, an equivalent deformation will be generated in both methods.

As mentioned above, it is difficult to practically employ this huge voltage, but it can be employed in FEM with a single direction of polarization which is much simpler and more economical than modeling the physical configuration of MFC with IDEs and opposite direction of polarization. The main contribution of this paper is the realization that the restrictions imposed in practice should not also be restrictions in modeling, which may require a very costly fine mesh resulting from imposing voltage differences at a spacing of 0.5 mm over the longitudinal length of MFC.

In conclusion, a simple finite element model of MFC can be developed on the basis of just two voltage boundary conditions applied at the actuator’s longitudinal extremes, thus permitting the usage of a considerable coarse mesh disregarding the physical structure of IDEs. This will significantly reduce the simulation time and enhance the computational cost while producing precise results, as demonstrated in the next section. Additionally, this approach allows us to model large numbers of MFC actuators bonded on the elastic morphing wing, as discussed in Section 7.

### 3.3. Modeling of MFC with 45° Fiber Orientation

**Figure 4:**
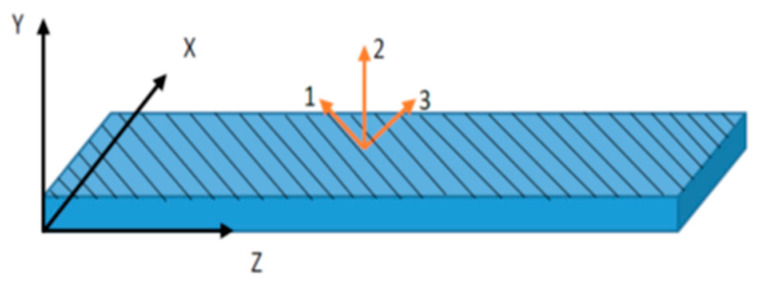
Schematic for MFC-F1 type with 45° fiber laminates in the 3-axis and IDE in the 1-axis.

The macro fiber composite actuator with 45° fiber orientation (F1 type) can produce a twisting motion with its d_33_ effect. This special type of MFC actuator has principal material coordinates in the O_123_ coordinates, as shown in Figure 4, where the direction of the fibers is aligned with coordinate 3 and the interdigitated electrodes are aligned with coordinate 1. The MFC has a body coordinate system (*x*, *y*, *z*) that needs to be related to the principal material coordinate system (1, 2, 3). Thus, the stresses and strains must be transformed in space between principal material coordinates and body coordinates. This transformation does not affect the material properties; rather, it is an equivalent expression for the stress directions after a certain rotation.

The three-dimensional stress transformation can be expressed as follows [48]:

(12) $$\left[\sigma^{\prime }\right]=\left[q\right]\left[\sigma \right]{\left[q\right]}^{T},$$

where

$$\;\left[\sigma \right]=\left[\begin{array}{ccc} \sigma_{x} & \tau_{\mathrm{xy}} & \tau_{\mathrm{xz}}\\ \tau_{\mathrm{xy}} & \sigma_{y} & \tau_{\mathrm{yz}}\\ \tau_{\mathrm{xz}} & \tau_{\mathrm{yz}} & \sigma_{z} \end{array}\right],\;\left[\sigma^{\prime }\right]=\left[\begin{array}{ccc} \sigma_{1} & \tau_{12} & \tau_{13}\\ \tau_{12} & \sigma_{2} & \tau_{23}\\ \tau_{13} & \tau_{23} & \sigma_{3} \end{array}\right],\;\mathrm{and}\;\left[q\right]=\left[\begin{array}{ccc} \cos\theta & 0 & -\sin\theta \\ 0 & 1 & 0\\ \sin\theta & 0 & \cos\theta \end{array}\right].$$

The coordinate system (*x*, *y*, *z*) is rotated by angle θ (equal to 45° in MFC-F1 type) around the *y*-coordinate to create the new coordinate system (1, 2, 3), as shown in Figure 4, where the 3-axis is in the direction of the fibers, the 1-axis system is in the interdigitated electrode direction, and the *y*-axis is aligned with the 2-axis.

The transformed stress matrix in the coordinate system (1, 2, 3) can be expressed in terms of the old stress matrix in the coordinate system (*x*, *y*, *z*) by a transformation matrix called [T] as follows:

(13) $$\left[\begin{array}{c} \sigma_{1}\\ \sigma_{2}\\ \sigma_{3}\\ \tau_{12}\\ \tau_{23}\\ \tau_{13} \end{array}\right]=\left[T\right]\left[\begin{array}{c} \sigma_{x}\\ \sigma_{y}\\ \sigma_{z}\\ \tau_{\mathrm{xy}}\\ \tau_{\mathrm{yz}}\\ \tau_{\mathrm{xz}} \end{array}\right],$$

where the transformation matrix [T] can be written as

(14) $$\left[T\right]=\left[\begin{array}{cccccc} \cos^{2}\;\theta & 0 & \sin^{2}\;\theta & 0 & 0 & -\sin2\theta \\ 0 & 1 & 0 & 0 & 0 & 0\\ \sin^{2}\;\theta & 0 & \cos^{2}\;\theta & 0 & 0 & \sin2\theta \\ 0 & 0 & 0 & \cos\theta & -\sin\theta & 0\\ 0 & 0 & 0 & \sin\theta & \cos\theta & 0\\ \frac{1}{2}\sin2\theta & 0 & -\frac{1}{2}\sin2\theta & 0 & 0 & \cos2\theta \end{array}\right].$$

It is worth mentioning that the above matrix is written in ANSYS format. The material properties of MFC in the principal material coordinate system (1, 2, 3) are known, and we need to find their equivalent behavior in the body coordinate system (*x*, *y*, *z*).

The strains in the coordinate system (*x*, *y*, *z*) can be expressed in terms of the coordinate system (1, 2, 3) by the same transformation matrix [T]. However, it uses the tensor shear strain definition, which is half the engineering shear strain [49], expressed as

(15) $$\left[\begin{array}{c} \varepsilon_{x}\\ \varepsilon_{y}\\ \varepsilon_{z}\\ \frac{\gamma_{\mathrm{xy}}}{2}\\ \frac{\gamma_{\mathrm{yz}}}{2}\\ \frac{\gamma_{\mathrm{xz}}}{2} \end{array}\right]={\left[T\right]}^{-1}\left[\begin{array}{c} \varepsilon_{1}\\ \varepsilon_{2}\\ \varepsilon_{3}\\ \frac{\gamma_{12}}{2}\\ \frac{\gamma_{23}}{2}\\ \frac{\gamma_{13}}{2} \end{array}\right]$$

The engineering shear strain vectors can be used instead of the tensor shear strain vectors by using Reuter’s transformation matrix [R] [50]. Moreover, the stress vector is related to the strain vector by the stiffness matrix [C]. Hence, the stress vector can be related to the strain vector in the body coordinate system (*x*, *y*, *z*) by a transformed stiffness matrix called $\bar{\left[C\right]}$.

(16) $$\left[\begin{array}{c} \sigma_{x}\\ \sigma_{y}\\ \sigma_{z}\\ \tau_{\mathrm{xy}}\\ \tau_{\mathrm{yz}}\\ \tau_{\mathrm{xz}} \end{array}\right]=\bar{\left[C\right]}\left[\begin{array}{c} \varepsilon_{x}\\ \varepsilon_{y}\\ \varepsilon_{z}\\ \gamma_{\mathrm{xy}}\\ \gamma_{\mathrm{yz}}\\ \gamma_{\mathrm{xz}} \end{array}\right]$$

Additionally, the transformed stiffness matrix $\bar{\left[C\right]}$ is expressed as

(17) $$\bar{\left[C\right]}={\left[T\right]}^{-1}\left[C\right]{\left[T\right]}^{-T}$$

where ${\left[T\right]}^{-T}=\left[R\right]\left[T\right]{\left[R\right]}^{-1}$, and *^T^* denotes matrix transpose.

Similarly, the transformed compliance matrix is expressed as

(18) $$\bar{\left[S\right]}={\bar{\left[C\right]}}^{-1}={\left[T\right]}^{T}{\left[C\right]}^{-1}\left[T\right]={\left[T\right]}^{T}\left[S\right]\left[T\right]$$

The previous derivation is for the mechanical behavior of the piezoelectric MFC actuator. Now, the electromechanical behavior of MFC can be investigated. The total strain produced in MFC is the sum of the mechanical strains generated from mechanical loads and controllable strains induced due to the employed electric field. The total strains produced in the principal material coordinate system (1, 2, 3) are

(19) $$\left[\begin{array}{c} \varepsilon_{1}\\ \varepsilon_{2}\\ \varepsilon_{3}\\ \gamma_{12}\\ \gamma_{23}\\ \gamma_{13} \end{array}\right]=\left[S\right]\left[\begin{array}{c} \sigma_{1}\\ \sigma_{2}\\ \sigma_{3}\\ \tau_{12}\\ \tau_{23}\\ \tau_{13} \end{array}\right]+\left[d\right]\left[\begin{array}{c} E_{1}\\ E_{2}\\ E_{3} \end{array}\right]$$

The transformed compliance matrix [S] is determined from the above derivation, which allows the transformed piezoelectric constants $\bar{\left[d\right]}$ to be deduced. The free strains induced due to the applied electric field in the principal material coordinate system (1, 2, 3) are

(20) $$\left[\begin{array}{c} \varepsilon_{1}\\ \varepsilon_{2}\\ \varepsilon_{3}\\ \gamma_{12}\\ \gamma_{23}\\ \gamma_{13} \end{array}\right]=\left[d\right]\left[\begin{array}{c} E_{1}\\ E_{2}\\ E_{3} \end{array}\right]$$

and the strains in the coordinate system (1, 2, 3) expressed in terms of the coordinate system (*x*, *y*, *z*) are

(21) $$\left[\begin{array}{c} \varepsilon_{1}\\ \varepsilon_{2}\\ \varepsilon_{3}\\ \gamma_{12}\\ \gamma_{23}\\ \gamma_{13} \end{array}\right]=\left[R\right]\left[T\right]{\left[R\right]}^{-1}\left[\begin{array}{c} \varepsilon_{x}\\ \varepsilon_{y}\\ \varepsilon_{z}\\ \gamma_{\mathrm{xy}}\\ \gamma_{\mathrm{yz}}\\ \gamma_{\mathrm{xz}} \end{array}\right]={\left[T\right]}^{-T}\left[\begin{array}{c} \varepsilon_{x}\\ \varepsilon_{y}\\ \varepsilon_{z}\\ \gamma_{\mathrm{xy}}\\ \gamma_{\mathrm{yz}}\\ \gamma_{\mathrm{xz}} \end{array}\right]$$

and the electric fields in the coordinate system (1, 2, 3) are transformed to the coordinate system (*x*, *y*, *z*) by the matrix [q] such that

(22) $$\left[\begin{array}{c} E_{1}\\ E_{2}\\ E_{3} \end{array}\right]=\left[q\right]\left[\begin{array}{c} E_{x}\\ E_{y}\\ E_{z} \end{array}\right]$$

Then, the equivalent free strains in the coordinate system (*x*, *y*, *z*) are

(23) $$\left[\begin{array}{c} \varepsilon_{x}\\ \varepsilon_{y}\\ \varepsilon_{z}\\ \gamma_{\mathrm{xy}}\\ \gamma_{\mathrm{yz}}\\ \gamma_{\mathrm{xz}} \end{array}\right]={\left[T\right]}^{T}\left[\begin{array}{c} \varepsilon_{1}\\ \varepsilon_{2}\\ \varepsilon_{3}\\ \gamma_{12}\\ \gamma_{23}\\ \gamma_{13} \end{array}\right]={\left[T\right]}^{T}\left[d\right]\left[\begin{array}{c} E_{1}\\ E_{2}\\ E_{3} \end{array}\right]={\left[T\right]}^{T}\left[d\right]\left[q\right]\left[\begin{array}{c} E_{x}\\ E_{y}\\ E_{z} \end{array}\right]$$

The electric fields in MFC actuators are always applied in the direction of the fibers only, which means that only $E_{3}$ has value and $E_{1}$=$\;E_{2}$ = 0. Let the electric field in the direction of the fibers be $E_{3}=E$, where E is a constant real value for the electric field between every electrode. In the coordinate system (*x*, *y*, *z*), $E_{3}$ will have two components in the *x*- and *z*-coordinates such that $E_{x}=E_{z}=E\cos45$. However, to apply the same technique as the MFC-P1 type, it is required to apply the electric field in the *z*-direction only. To achieve this, we pre-multiply the electric vector [E_x_ E_y_ E_z_]*^T^* in Equation (23) by [q]^−1^ [q], as shown in Equation (24).

(24) $$\left[\begin{array}{c} \varepsilon_{x}\\ \varepsilon_{y}\\ \varepsilon_{z}\\ \gamma_{\mathrm{xy}}\\ \gamma_{\mathrm{yz}}\\ \gamma_{\mathrm{xz}} \end{array}\right]={\left[T\right]}^{T}\left[d\right]\left[q\right]{\left[q\right]}^{-1}\left[q\right]\left[\begin{array}{c} E\cos45\\ 0\\ E\cos45 \end{array}\right]={\left[T\right]}^{T}\left[d\right]\left[\begin{array}{c} 0\\ 0\\ E \end{array}\right]=\bar{\left[d\right]}\left[\begin{array}{c} 0\\ 0\\ E \end{array}\right]$$

Therefore, as Equation (24) indicates, we are now working with an equivalent system that gives the same strain as the original system by applying the electric field in the *z*-direction instead of the 3-direction. The equivalent system has modified piezoelectric constants $\bar{\left[d\right]}={\left[T\right]}^{T}\left[d\right]$. Now, the MFC-F1 type with 45° fiber orientation can be modeled using the same technique as the MFC-P1 type.

## 4. Simulation Validation of the Proposed Technique

### 4.1. Validation of the Proposed Technique with the Detailed FE Model of MFC with 45° Fiber Orientation

**Figure 5:**
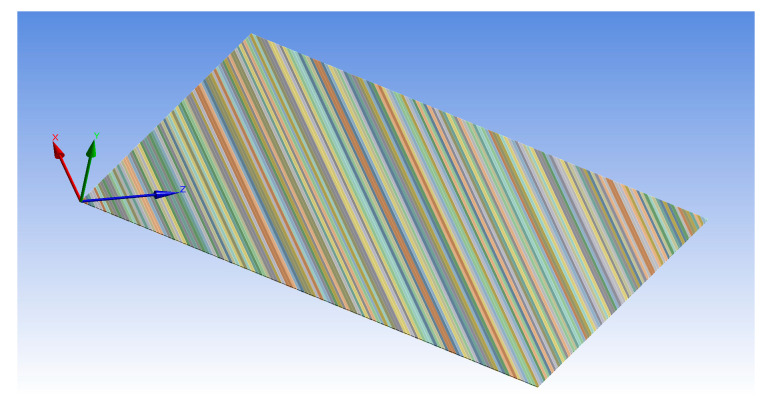
Detailed FE model for MFC-F1 type with 45° fibers orientation.

A finite element model of MFC was developed for MFC-8557-F1 with IDEs and opposite pooling directions, where the fiber direction was oriented by 45° from the longitudinal direction of the MFC body, as displayed in Figure 5. The detailed model of the MFC-F1 type was verified by comparing its free strain with that of the reference-free strain stated in the datasheet. The model of MFC was divided into 201 segments of piezoelectric material along the fiber direction to satisfy the electrode spacing of 0.5 mm. This model was supported as a cantilever and meshed using 66,323 elements. The coordinate system of the FE model was oriented such that its *z*-axis made an angle of 45° to the longitudinal direction of the MFC body, as displayed in Figure 5. A voltage difference was employed over each segment along the oriented *z*-axis. The simulation was conducted to check the peak-to-peak free strain in the oriented *z*-axis and was compared with the reference value in the datasheet [4] (i.e., 1750 ppm).

**Figure 6:**
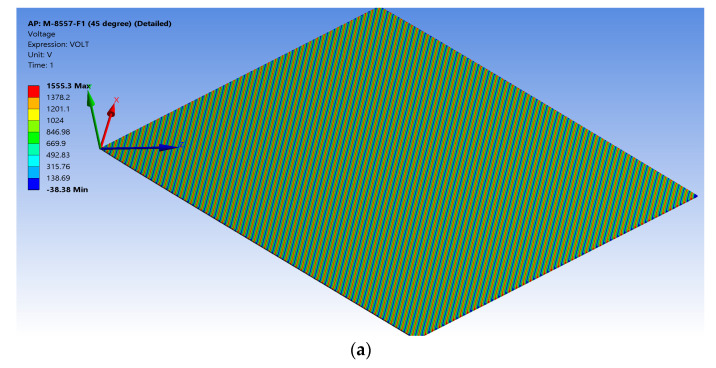

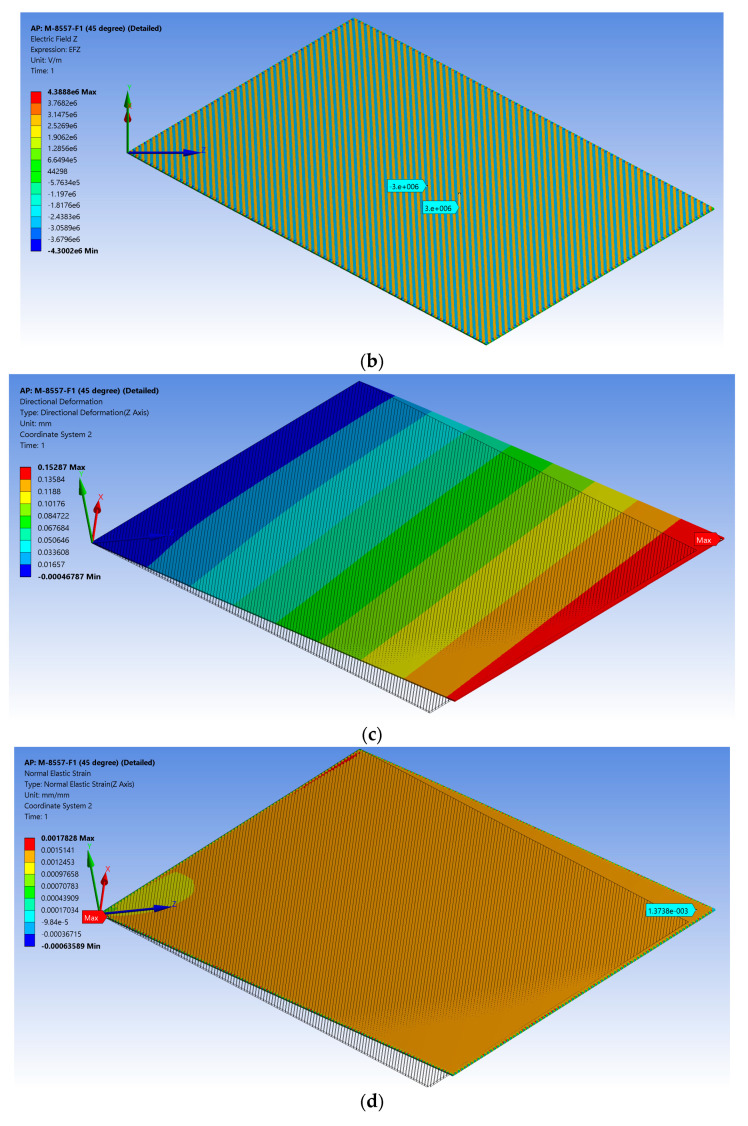

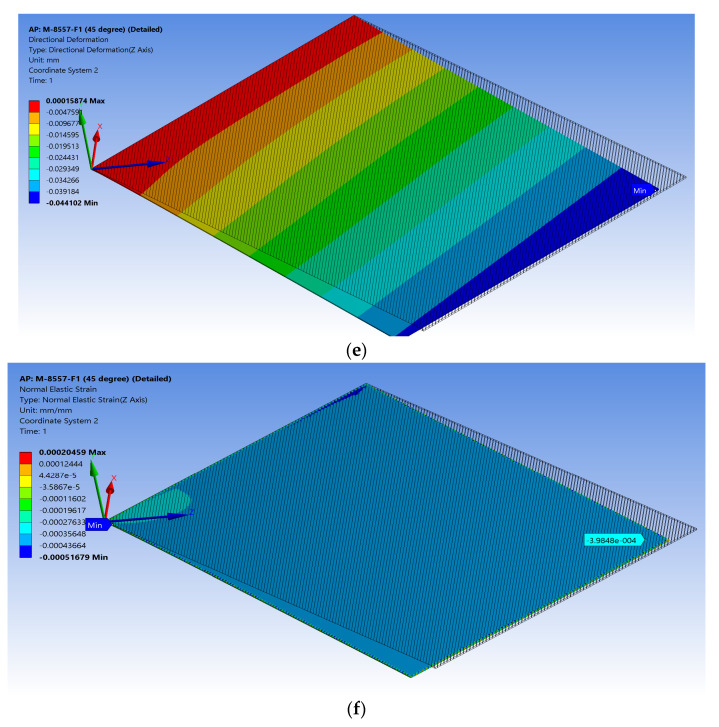
Detailed FE model of M-8557-F1 showing (**a**) applied voltage difference of 1500 V for every segment, (**b**) the generated electric field of about ±3 × 10^6^ V/m, (**c**) the result for elongation displacement in the *z*-axis, (**d**) the result for max elongation strain, (**e**) the result for compressive displacement in the *z*-axis, and (**f**) the result for max compressive strain.

This simulation was performed by applying a maximum voltage difference of +1500 V, as shown in Figure 6a, which resulted in generating high electric fields of about ±3 × 10^6^ V/m, as shown in Figure 6b. The MFC had a maximum elongation displacement of 0.15287 mm, as shown in Figure 6c, and a maximum elongation strain of about 1373.8 ppm, as shown in Figure 6d. Then, the simulation was performed under a voltage difference of −500 V (low electric field), which resulted in a maximum compressive displacement of −0.044102 mm, as shown in Figure 6e, and maximum compressive strain of about −398.48 ppm, as shown in Figure 6f. The peak-to-peak free strain resulting from this detailed model was about 1772.28 ppm with a percentage difference of about 1.27% compared to the reference value in the datasheet. The simulation had a memory usage of about 1 GB, a result file size of about 63 MB, and an elapsed simulation time of about 209 s.

**Figure 7:**
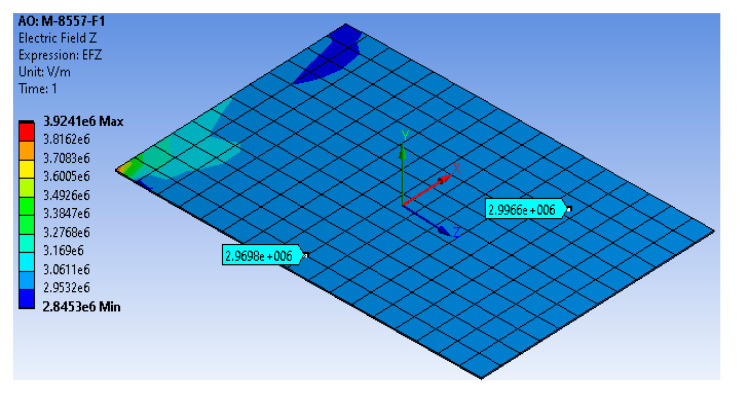
Simplified FE model of the electric field generated from the applied voltage difference of 255,000 V, which is equivalent to 1500 V in the physical MFC.

The FE model of the MFC-F1 type (M-8557-F1) was developed again using the newly proposed technique. The MFC was modeled as a simple monolithic piezoelectric material supplemented by an applied electric field in the longitudinal direction of the MFC body. The simplified FE model had only 204 elements, as shown in Figure 7 compared to 66,323 elements for the detailed FE model. The transformation of material properties between the principal material coordinates and body coordinates was used according to the derivation in the previous section. The transformed compliance matrix and the modified piezoelectric constants were $\bar{\left[S\right]}={\left[T\right]}^{T}\left[S\right]\left[T\right]$ and $\bar{\left[d\right]}={\left[T\right]}^{T}\left[d\right]$, respectively.

**Figure 8:**
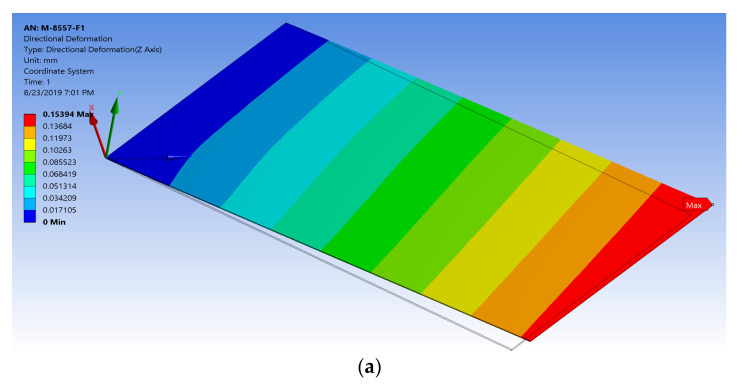

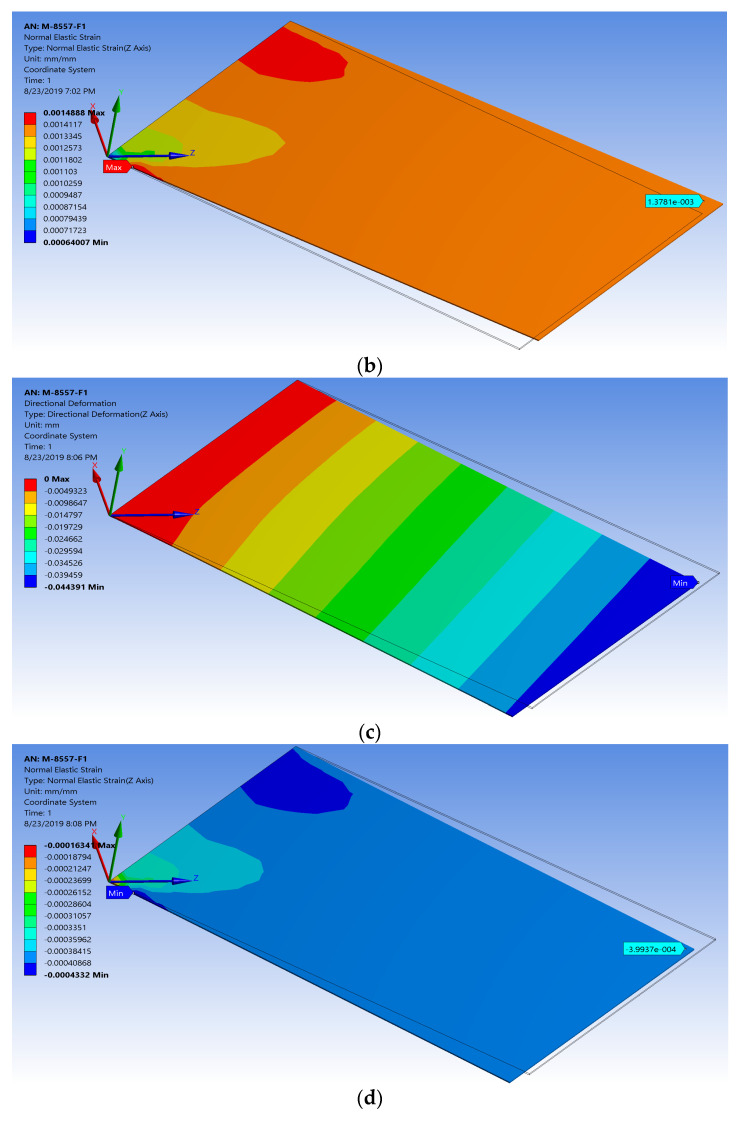
Simplified FE model of M-8557-F1 showing (**a**) the result for elongation displacement in the *z*-axis, (**b**) the result for max elongation strain, (**c**) the result for compressive displacement in the *z*-axis, and (**d**) the result for max compressive strain.

Similarly, the simulation was conducted for maximum elongation/compressive strains. A high electric field was applied to the FE model (+3 × 10^6^ V/m), corresponding to a new applied voltage difference of 255,000 V in the proposed technique. The model generated almost the same high electric field as that generated from the detailed model with a percentage difference of 1%, as shown in Figure 7. Furthermore, the simplified FE model produced a maximum elongation displacement of 0.15394 mm, as shown in Figure 8a, with a percentage difference of about 0.7% from the detailed MFC model and maximum elongation strain of 1378.1 ppm, as shown in Figure 8b, with a percentage difference of about 0.3% from the detailed MFC model. For the low electric field (−500 V), the model produced a maximum compressive displacement of −0.044391 mm, as shown in Figure 8c, with a percentage difference of about 0.65% from the detailed MFC model and maximum compressive strain of −399.37 ppm, as shown in Figure 8d, with a percentage difference of about 0.2% from the detailed MFC model. Thus, the peak-to-peak free strain resulting from this simplified model with the newly proposed technique was about 1777.47 ppm with a percentage difference of about 0.3% from the detailed model and with a percentage difference of about 1.6% from the reference value in the datasheet. The simulation had a memory usage of about 262 MB, a result file size of about 704 KB, and an elapsed simulation time of about 3 s.

The comparison between the simplified FE model of MFC and its equivalent detailed model is summarized in Table 3. The computational costs for the simplified model compared with the detailed model were reduced by about 74% for memory usage, 99% for result file size, and 98.6% for elapsed simulation time. It is worth mentioning that these computational costs resulted from simulating only one MFC. This enhancement in the computational costs will be more effective when simulating a large number of MFCs.

### 4.2. Validation of the Proposed Technique for Bimorph Wing

The developed technique for modeling MFC with 45° fiber orientation was applied for FE modeling of a morphing wing. The proposed FE model was validated by the results of Wang’s work in [24,25] for FE modeling of a morphing wing with bimorph configuration. Bending and torsional active shapes are needed for morphing wings to obtain desired aerodynamic behaviors. Whereas the bending shape interacts with aerodynamic loads and affects the overall flight conditions, a twisting motion can change the wing’s angle of attack so that the desired lifting aerodynamic behaviors can be achieved. Both bending and torsional active shapes can be obtained using MFC actuators with 45° fiber orientation.

**Figure 9:**
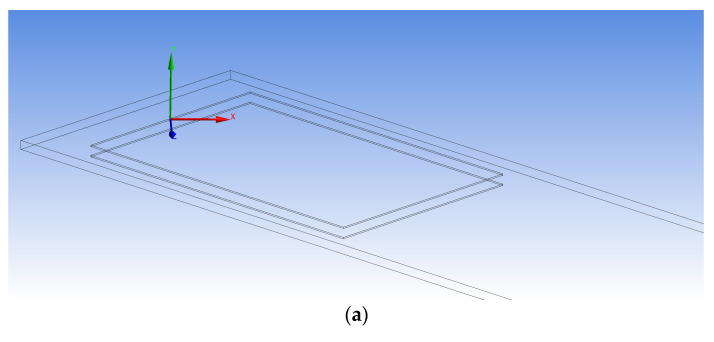

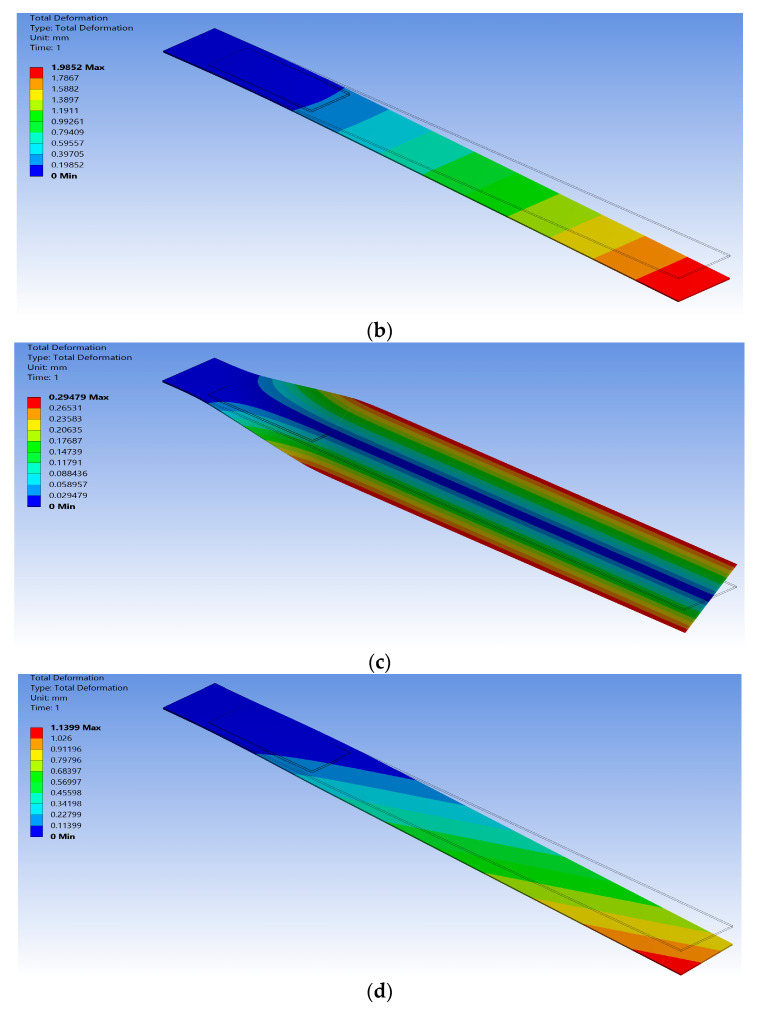
FE model of morphing wing with bimorph configuration of MFC actuators. (**a**) Two MFCs are bonded on the top and bottom surfaces of the cantilever beam. (**b**) The bending moment resulting from applying different voltages on MFCs. (**c**) The torsion moment resulting from applying the same voltages on MFCs. (**d**) The coupled torsional and bending moments resulting from actuating only one MFC from either side.

An FE model was developed consisting of a cantilever beam acting as a wing substrate and two MFC actuators bonded on the upper and lower surfaces of the beam, as displayed in Figure 9a. The top MFC actuator had a −45° orientation from the longitudinal direction, while the bottom MFC actuator had a 45° orientation from the longitudinal direction. The same dimensions in the setup of [24,25] were used to develop the morphing wing model. The newly proposed technique conducted in this section for FE modeling of MFC with 45° fiber orientation was applied in this simulation. The torsional shape could be obtained by applying the same voltage differences of 500 V to the two MFCs. The bending shape could be obtained by applying 500 V to one MFC while applying −500 V to the other MFC [24,25].

The active length for the MFC used in the FE model was 120 mm. In our proposed technique, we used a voltage difference of 120,000 V, corresponding to the applied voltage difference of 500 V in the physical MFC. The maximum deflections resulting from Wang’s work for bending and torsional configurations were about 2 mm and 0.3 mm, respectively [24,25]. The maximum deflection resulting from our FE model with the newly proposed technique for bending configuration was 1.9852 mm, as shown in Figure 9b, with a percentage difference of about 0.7% from Wang’s work. On the other hand, the maximum deflection resulting from our FE model with the newly proposed technique for torsional configuration was 0.29479 mm, as shown in Figure 9c, with a percentage difference of about 1.7% from Wang’s work. Moreover, a coupled shape of bending and torsion moments could be achieved by actuating only one actuator, as shown in Figure 9d.

## 5. Experimental Verification of the Proposed Technique

This section presents the experimental works carried out to verify the proposed technique of modeling MFC. A simple low-cost prototype was designed and implemented to perform the experimental verification. The prototype consisted of the following: cantilever beam, macro fiber composite piezoelectric material, setup frame, strain sensors with ADC converter, Arduino Mega 2560, and power amplifier with boost converter circuit.

A thin beam with a rectangular cross-section was manufactured using a laser cutting machine. This beam had a length of 230 mm, a width of 42 mm, and a thickness of 0.5 mm. The beam was created from aluminum, Al-1050, with a density of 2710 kg/m^3^, Young’s modulus of 71 GPa, Poisson’s ratio of 0.33, and shear modulus of 26.7 GPa. In the experiment, the overhanging length of the beam was 200 mm, with 30 mm fully fixed using a mini vice.

The piezo composite material employed in this verification was macro fiber composite (MFC). The MFC was coded as M-8528-F1, indicating an active length of 85 mm, active width of 28 mm, and 45° fiber orientation. This piezo-composite material had an overall length of 105 mm, overall width of 35 mm, and thickness of 0.3 mm. The MFC actuator was positioned on the surface of the beam at 50% of the beam length. An acrylic frame was constructed using Autodesk Inventor and manufactured by a laser cutting machine. This frame was used to hold the mini vice with the cantilever beam, micrometer, and electric circuits.

The strain sensors used in the experiment were strain gauges BF-350 with a nominal resistance of 350 Ω, gauge grid dimensions of 3.2 mm × 3.1 mm, and gauge packing dimensions of 7.4 mm × 4.4 mm. The strain gauges were bonded symmetrically on the top and bottom surfaces of the beam.

The MFC used in the experiment produced a combined load of twisting and bending moments on the beam. The default operation of the strain gauge was to measure axial strain on a surface under bending moment only. To measure the combined load on the cantilever beam, the strain gauges were operated in a rectangular rosette (0/45°/90°) configuration. This configuration measured three different strains at three different angles. Through mathematical equations, maximum/minimum principal strain/stress, shear strain, and principal angle could be deduced at a specific point on the cantilever beam.

The strain gauges on the upper and bottom surfaces were connected to three half Wheatstone bridges. These bridges were implemented on two breadboards and connected to the strain gauges using shielded wires to attenuate noises on acquired signals. Each bridge was completed by two equal resistors of 470 Ω. The strains generated on the gauges were calculated from the half-bridge using the following relationship:

(25) $$V_{\mathrm{out}}=2\times \frac{1}{4}\times K\times \varepsilon \times V_{\mathrm{in}}$$

where $V_{\mathrm{out}}$ is the output voltage from the bridge, $V_{\mathrm{in}}$ is the input voltage to the bridge, K is the gauge factor (K = 2), and ε is the strain generated on the gauge.

HX711 is a precision 24 bit analog-to-digital converter (ADC) designed to be used with strain gauges. This ADC was used in this test to convert the analog signals from sensor bridges to digital signals sent to the Arduino microcontroller. The full scale of input voltage to the ADC was ±20 mV, represented by a decimal number of 8,388,608 (2^23^). Furthermore, this ADC had an on-chip active low noise programmable gain amplifier (PGA) which was set to 128 in this experiment. The Arduino microcontroller supplied the HX711 circuit with 5 V. The HX711 circuit sent about 4 V to the sensor bridge as an excitation signal. Despite using low-noise PGA on the chip of HX711, there was still noise generated from this circuit. A physical low-pass filter using an RC-circuit and a programmable low-pass filter and/or median filter were used to attenuate those noises.

Arduino Mega 2560 was used in this experiment as a data acquisition unit. The data collected by Arduino were sent to the computer via a USB serial port. The Arduino was programmed by C-language from the Arduino IDE software. In this experiment, Arduino read data from HX711, representing the strains generated on the gauges. Additionally, it was used to send a PWM signal to the boost converter circuit to control its duty cycle.

### 5.1. Mathematical Equations of Rectangular Rosette

**Figure 10:**
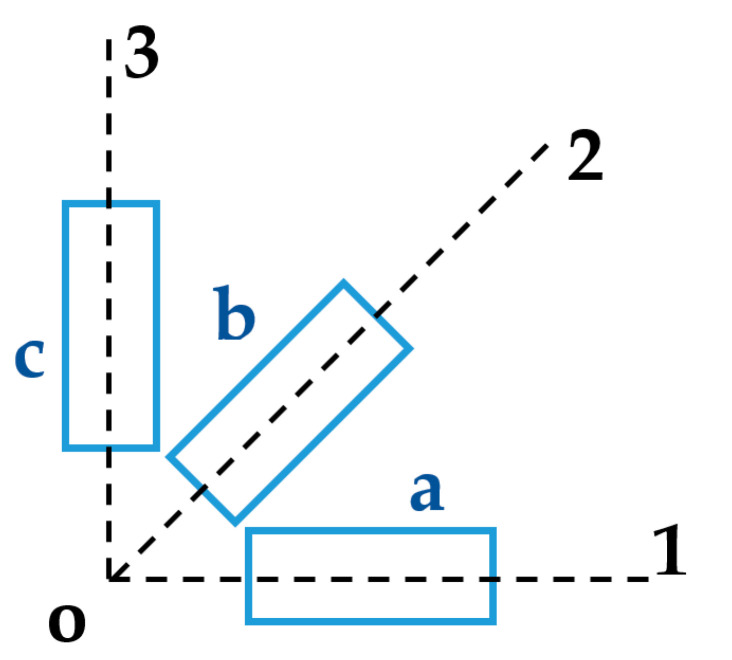
Schematic showing three strain gauges (a, b, c) aligned with three axes (1, 2, 3).

The three strain gauges (a, b, and c) were aligned with the three axes (1, 2, and 3). The three axes met at a reference point “o”, as illustrated in Figure 10. The three strain gauges (a, b, and c) were located at 0°, 45°, and 90°, respectively.

The following equations were applied at the reference point:

(26) $$\varepsilon_{a}=\varepsilon_{1}\cos^{2}\theta_{a}+\varepsilon_{3}\sin^{2}\theta_{a}+\gamma_{13}\sin\theta_{a}\cos\theta_{a}$$

(27) $$\varepsilon_{b}=\varepsilon_{1}\cos^{2}\theta_{b}+\varepsilon_{3}\sin^{2}\theta_{b}+\gamma_{13}\sin\theta_{b}\cos\theta_{b}$$

(28) $$\varepsilon_{c}=\varepsilon_{1}\cos^{2}\theta_{c}+\varepsilon_{3}\sin^{2}\theta_{c}+\gamma_{13}\sin\theta_{c}\cos\theta_{c}$$

where $\varepsilon_{a}$, $\varepsilon_{b}$, and $\varepsilon_{c}$ are axial strains produced on strain gauges a, b, and c, respectively. $\varepsilon_{1}$ and $\varepsilon_{3}$ are axial strains on axes 1 and 3, respectively. $\gamma_{13}$ is the shear strain in the 1–3 plane. $\theta_{a}$, $\theta_{b}$, and $\theta_{c}$ are angles of the three strain gauges from the 1-axis, such that $\theta_{a}=0{}^{\circ}$, $\theta_{b}=45{}^{\circ}$, and $\theta_{c}=90{}^{\circ}$.

After substituting the angles of the strain gauges, one can find that $\varepsilon_{1}=\varepsilon_{a}$, $\varepsilon_{3}=\varepsilon_{c}$, and $\gamma_{13}=2\varepsilon_{b}-\varepsilon_{a}-\varepsilon_{c}$.

Then, the principal strain, principal stress, and principal angle can be calculated from

(29) $$\varepsilon_{p}=\frac{1}{2}\left[\varepsilon_{1}+\varepsilon_{3}\pm \sqrt{{\left(\varepsilon_{1}-\varepsilon_{3}\right)}^{2}+\gamma_{13}{}^{2}}\right]$$

(30) $$\sigma_{p}=\frac{E}{2}\left[\frac{\varepsilon_{1}+\varepsilon_{3}}{1-\nu }\pm \frac{\sqrt{{\left(\varepsilon_{1}-\varepsilon_{3}\right)}^{2}+\gamma_{13}{}^{2}}}{1+\nu }\right]$$

(31) $$\theta_{p}=\frac{1}{2}\tan^{-1}\frac{\gamma_{13}}{\varepsilon_{1}-\varepsilon_{3}}$$

where $\varepsilon_{p}$, $\sigma_{p}$, and $\theta_{p}$ are the principal strain, principal stress, and principal angle, respectively.

### 5.2. Power Amplifier and Boost Converter Circuit

**Figure 11:**
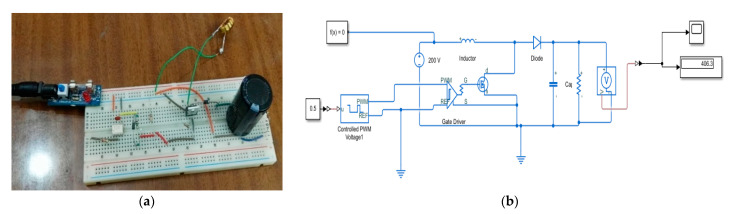
(**a**) Boost converter circuit; (**b**) MATLAB-Simscape model for the boost converter.

A power amplifier was required to actuate the MFC actuator which can be supplied from −500 V to 1500 V. In this experiment, a low-field mode was used to actuate MFC from −400 V to +400 V. A linear amplifier EPA-104 was used in the experiment to supply voltage within ±200 V. To supply voltage more than 200 V, a boost converter circuit was designed and implemented on a breadboard, as shown in Figure 11a. The electronic components of the boost converter circuit could withstand up to 450 V. Therefore, the boost converter was limited to boost the 200 V from the power amplifier up to 400 V. TLP-250 was used as a gate driver for the power MOSFET IRF-840 in the boost converter. The boost converter circuit was designed and simulated using MATLAB-Simscape, as shown in Figure 11b, before being implemented on the breadboard.

The relationship between input and output voltage of the boost converter can be described by the following equation:

(32) $$V_{\mathrm{out}}=\frac{V_{\mathrm{in}}}{1-D}$$

where D is the duty cycle set to about 50% in the experiment, $V_{\mathrm{in}}$ is the input voltage to the boost converter, which was supplied from the EPA-104 with 200 V, and $V_{\mathrm{out}}$ is the output voltage from the boost converter supplied to the MFC actuator.

### 5.3. Experimental Procedures

**Figure 12:**
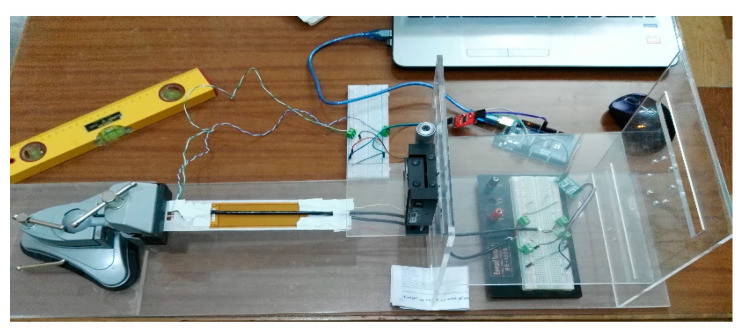
Prototype for the first verification.

**Figure 13:**
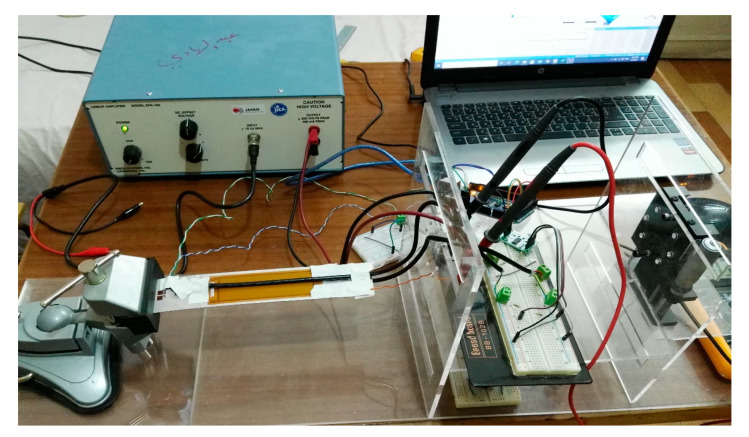
Prototype for the second verification.

- Firstly, the FE model was verified with applied displacements at the tip of the cantilever beam without activating MFC, as illustrated in Figure 12. The measurements were acquired from the rosette strain gauges and compared with their equivalent strains in the FE simulation.
- Secondly, the FE simulation was verified with the physical MFC placed on the beam, as illustrated in Figure 13. The MFC actuator was statically energized from 0 to 400 V with a step of 100 V. The measurements were acquired from the three strain gauges and compared with their equivalent strains in the FE simulation.
- It is worth mentioning that each reading needed some time to settle for every measurement. Moreover, there was a non-zero initial offset in the measurements which was compensated for by the readings. This offset was produced from the HX711 circuit, bridge circuit, and the extra weight on the beam due to wires. Additionally, the offset may vary for each set of measurements, whereas, if the wires are slightly moved, the readings will also change. Thus, the non-zero offset was checked frequently for every set of measurements.

## 6. Experimental Results and Discussion

### 6.1. Verification of the FE Simulation under Tip Displacements

**Figure 14:**
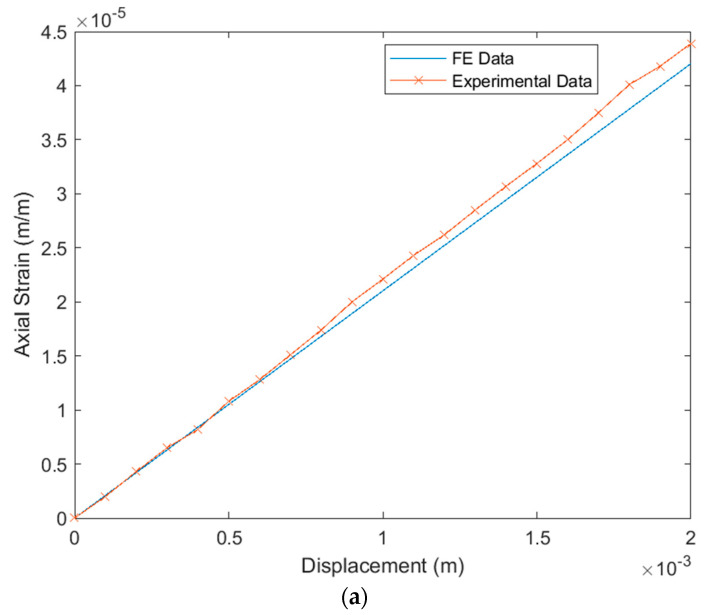

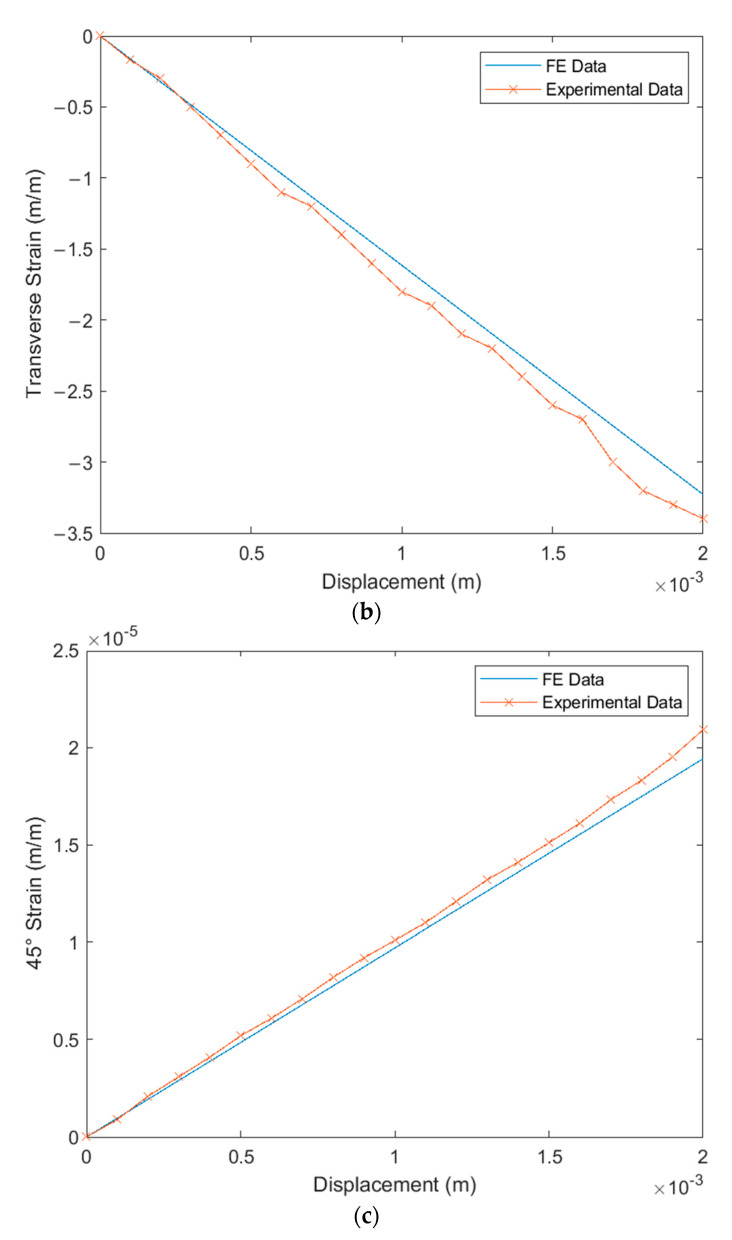
Verification of FE simulation strains (m/m) and experimental strains (m/m) due to tip displacements (µm): (**a**) axial strain (ε_c_); (**b**) transverse strain (ε_a_); (**c**) strain at 45° (ε_b_).

The FE simulation was compared to the experimental results acquired from the rosette strain gauges under static displacements at the tip of the cantilever beam. This comparison is demonstrated in Figure 14a for axial strain, Figure 14b for transverse strain, and Figure 14c for strain at 45°.The root-mean-square errors (RMSEs) between simulation and experiment were 1.14 × 10^−6^ (m/m) for axial strain, 1.47 × 10^−7^ (m/m) for transverse strain, and 5.85 × 10^−7^ (m/m) for strain a 45°. The maximum percentage differences between simulation and experiment were 6% for axial strain at 1800 µm, 13.6% for transverse strain at 600 µm, and 7.8% for strain at 45° at 100 µm.

### 6.2. Verification of FE Simulation under MFC Actuation

**Figure 15:**
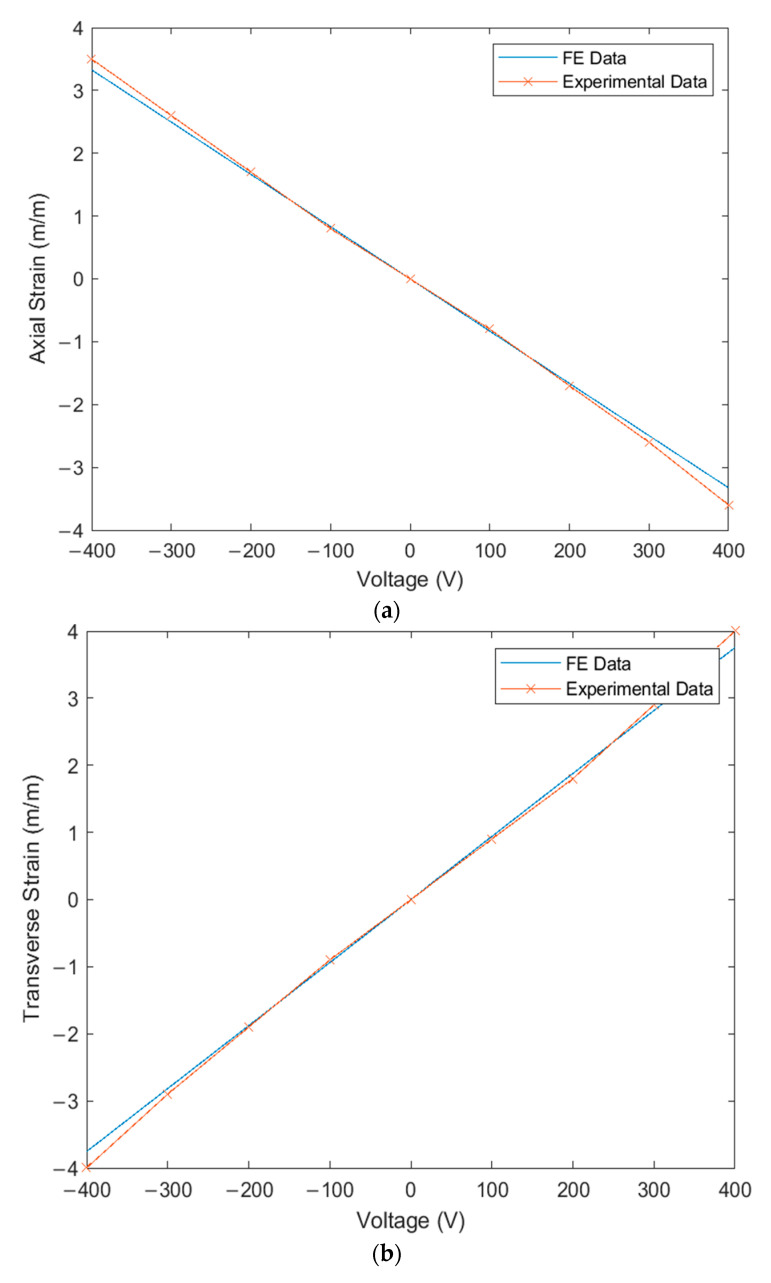

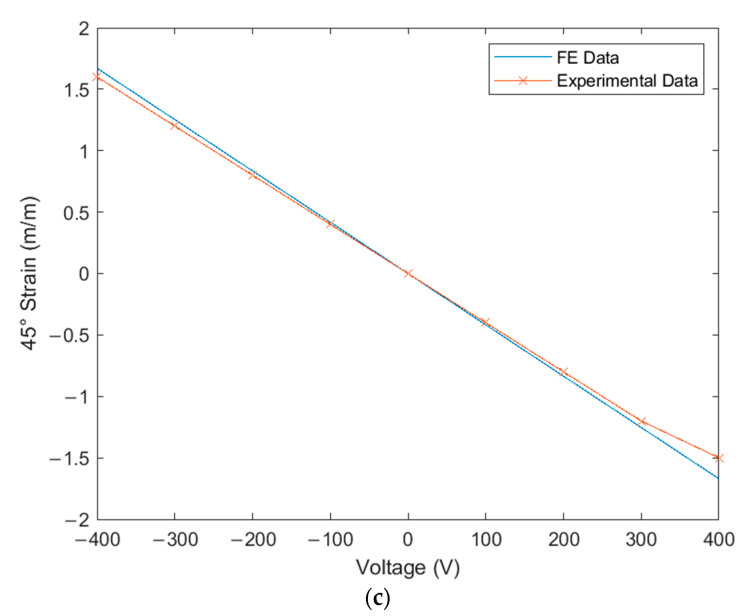
Verification of FE simulation strains (m/m) and experimental strains (m/m) due to MFC actuation in volts (V): (**a**) axial strain (ε_c_); (**b**) transverse strain (ε_a_); (**c**) strain at 45° (ε_b_).

The FE simulation of the proposed technique for modeling MFC was compared to the experimental results acquired from the rosette strain gauges under static load from the MFC actuator. This comparison is illustrated in Figure 15a for axial strain, Figure 15b for transverse strain, and Figure 15c for strain at 45°. The RMSEs between simulation and experiment were 1.21 × 10^−8^ (m/m) for axial strain, 1.26 × 10^−8^ (m/m) for transverse strain, and 6.9 × 10^−9^ (m/m) for strain at 45°. The maximum percentage differences between simulation and experiment were 8.2% for axial strain at 400 V, 6.5% for transverse strain at 400 V, and 10.2% for strain at 45° at 400 V.

The shear strain, maximum and minimum principal strains, and principal angle could be produced from the acquired axial, transverse, and 45° strains. Table 4 illustrates the comparison between simulation and experiment due to applied voltage on the MFC actuator. The RMSEs between simulation and experiment were 1.56 × 10^−8^ (m/m) for shear strain, 8.02 × 10^−9^ (m/m) for maximum principal strain, 9.19 × 10^−9^ (m/m) for minimum principal strain, and 0.95° for principal angle. The maximum percentage differences between simulation and experiment were 9.8% for shear strain at 400 V, 5.6% for maximum principal strain at 200 V, −4.8% for minimum principal strain at −400 V, and 14% for the principal angle at 400 V. The experimental results successfully verify the proposed technique of modeling MFC.

## 7. Modeling of Morphing Wing Actuated by Multiple MFCs Using the Newly Proposed Technique

**Figure 16:**
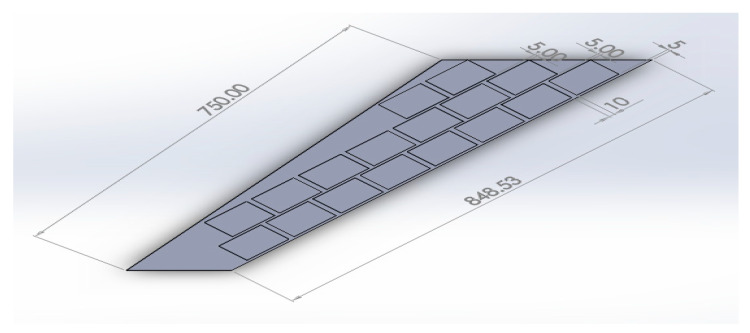
CAD model for a sweptback wing with MFC actuators on its surface (dimensions are in mm).

To show the benefits of the conducted work, the newly proposed technique for FE modeling of MFC was used to analyze a variety of active shapes for a morphing wing. These shapes are extremely costly to analyze using the detailed model of MFC. The FE model was developed for morphing a sweptback wing covered by many MFCs. The full dimensions for the sweptback wing are shown in the CAD model of Figure 16. The wing had a thickness of 2 mm, while each MFC had a thickness of 0.3 mm. The MFCs were distributed to cover the major area of the upper surface of the sweptback wing. The same distribution of MFCs existed on the bottom surface. According to the authors’ knowledge, no previous work has been conducted to simulate a morphing wing with a large number of MFC actuators.

**Figure 17:**
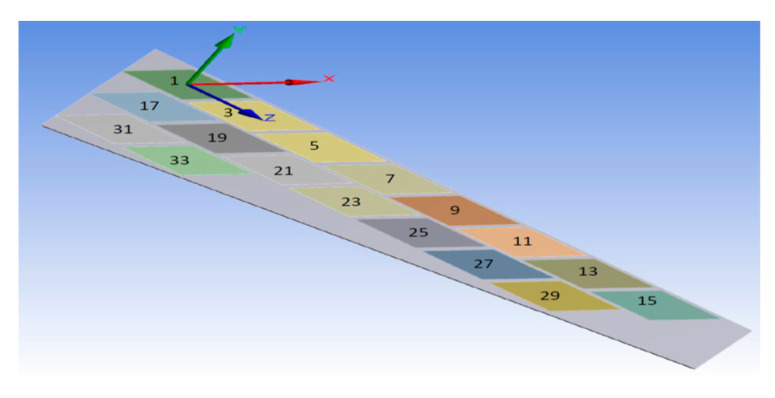
FE model of sweptback wing showing numbered MFC actuators and their coordinate system.

The FE model of the morphing wing had 34 MFC actuators distributed on both sides of the wing, as shown in Figure 17. The actuators were numbered from 1 to 34, where the odd numbers were given to MFC actuators on the upper surface and the even numbers were given to the actuators on the bottom surface, such that the two actuators occupying the same *xz*-area but on different sides of the wing had consecutive numbers. The MFC actuators (1 to 16) and (31 to 34) were M-8557-P1 type, which generated longitudinal strain along the *z*-axis, while the MFC actuators (17 to 30) were M-8557-F1 type, which generated strain along its 45° oriented fibers. The newly proposed technique of FE modeling for both MFC-P1 and MFC-F1 was applied in this simulation. The FE model had a total number of 4164 mesh elements. The FE model used quadratic-order mesh elements. The simulation for each morphing configuration had a computational time of about 5 s, 201 MB memory usage, and 3.7 MB result file size.

To demonstrate the capability of our approach, MFC-P1 type actuators were distributed on the morphing wing such that they produced bending deformation in the *x*-direction, while MFC-F1 type actuators were distributed such that they produced bending deformation in the *y*-direction and twisting moment around the *z*-axis.

Different configurations of morphing wing were successfully obtained by applying different voltages to MFCs, as illustrated in Table 5. This indicates the benefits of using a large number of MFCs, whether MFC-P1 type, MFC-F1 type, or their combination; consequently, the importance of the proposed modeling approaches was established in facilitating the analysis of this morphing wing with a large number of MFCs utilizing feasible computational power.

The results show that the proposed FE techniques can analyze efficiently (very low computational time and small memory usage) a variety of morphing wing shapes that are generated with a large number of MFC actuators. This variety of morphing wing shapes can greatly affect lift, pitch moment, and rolling moment, which increases the maneuverability of the associated air vehicle.

As a future work, an aeroelastic mathematical model can be developed for the presented morphing wing after applying aerodynamic loads. This model describes the inertial and elastic coupling among the wing structural dynamics, the dynamics of the developed simplified model of MFCs, and the aerodynamic behavior. It is to be noted that the proposed simplified technique employed in the mathematical model will be used to model MFC not only as an actuator but also as a sensor. Then, the mathematical model can be reduced easily, thanks to the developed simplified model of MFC, using the modal decomposition method to a reduced-order linear dynamic model for control purposes. Consequently, a control methodology can be implemented for the current morphing wing to allow a small UAV to perform specified flight maneuvers or track desired trajectories at fast response with low computational time.

## 8. Conclusions

An efficient technique was presented in this work to model an MFC actuator with its IDEs as a simple monolithic piezo-ceramic actuator. This technique presents a simplified FE model for the homogenized MFC actuator with only two electrodes at its longitudinal extremes instead of the current modeling using a physically large number of electrodes, which results in a very fine FE mesh with a high computational cost. The realization that the restrictions imposed in practice should not also be restrictions in modeling was the key point in developing the conducted technique. The new voltage proposed in this technique theoretically produces the same electric field, strain, and deformation as the physical MFC. The simulation accuracy was verified by comparing the simplified FE model with the detailed FE model, reference value in the datasheet, and previously published FE simulation of a morphing wing. Furthermore, experimental tests were conducted using a cantilever beam equipped with a physical MFC actuator. Both simulations and experimental results showed good consistency for the proposed technique of modeling MFC with low computational cost.

The proposed simplified FE model facilitates the modeling of smart structures with a large number of MFC actuators, which is not feasible using the previous detailed FE model. The proposed technique was successfully applied in modeling a morphing wing covered almost entirely with MFC actuators. A variety of wing shapes were successfully generated to achieve different aerodynamic characteristics with very low computational cost and memory usage.

## Figures and Tables

**Table 1:** Comparison between Williams’s experimental measurements and manufacturer datasheet.

	Williams’s Experimental Work [29]	Manufacturer Datasheet [4]
**E_3_ (GPa)**	29.4	30.336
**E_1_ (GPa)**	15.2	15.3857
$\nu_{31}$	0.312	0.31
$\nu_{32}$	0.161	0.16
**G_13_ (GPa)**	6.06	5.515

**Table 2:** Piezoelectric strain constants for high and low fields.

	$d_{33}\;(\mathrm{pm}/V)$	$d_{31}\;(\mathrm{pm}/V)$	$\mathrm{Free}\;\mathrm{Strain}\;\mathrm{per}\;\mathrm{Voltage}\;\mathrm{for}\;d_{33}\;(\mathrm{ppm}/V)$
**High field** (|E|>1 kV/mm)	460	−210	~0.9
**Low Field** (|E|<1 kV/mm)	400	−170	~0.75

**Table 3:** Comparison between simplified FE model and detailed FE model of MFC.

Simulation Parameters	Simple FE Model	Detailed FE Model	% Difference
Number of mesh elements	204 elements	66,323 elements	99.7%
Computational time	3 s	209 s	98.6%
Memory usage	262 MB	1 GB	74.4%
Result file size	407 KB	63 MB	99.4%

**Table 4:** Comparison between FE simulation and experimental results for applied voltage on MFC actuator.

Voltage (V)	% Difference in Shear Strain	% Difference in Maximum Principal Strain	% Difference in Minimum Principal Strain	% Difference in Principal Angle
−400	1.86%	−3.38%	−4.80%	6.31%
−300	4.51%	−2.15%	−1.33%	6.71%
−200	4.51%	−0.58%	0.09%	5.22%
−100	4.51%	4.10%	4.29%	0.46%
0	0.00%	0.00%	0.00%	0.00%
100	4.51%	4.29%	4.10%	0.46%
200	9.82%	5.62%	0.32%	7.57%
300	4.51%	−1.33%	−2.15%	6.71%
400	9.81%	−3.18%	−4.21%	14.00%

**Table 5:** Different configurations for morphing sweptback wing.

	Voltage Applied to MFC	Maximum Deflection in *x*-, *y*-, and *z*-Axes	Comments on the Deformation	Total Deformation
**1**	17 → 30 = −500 V	Δ*x* = −25.6 µm Δ*y* = −2.75 mm Δ*z* = −6.16 µm	All MFCs-F1 type are actuated to generate a clockwise twisting moment	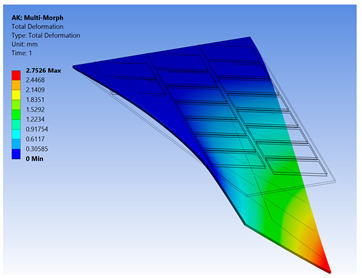
**2**	17 → 30 = +500 V	Δ*x* = 25.6 µm Δ*y* = 2.75 mm Δ*z* = 6.16 µm	All MFCs-F1 type are actuated to generate a counterclockwise twisting moment	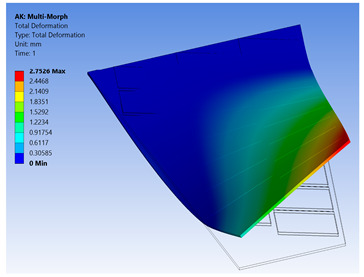
**3**	1 → 16 = +1500 V 31 → 34 = +1500 V	Δ*x* = −0.24 mm Δ*y* = −70 µm Δ*z* = 89.8 µm	All MFCs-P1 type are actuated to generate a bending moment	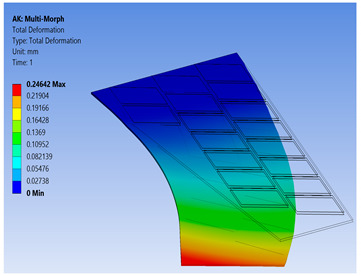
**4**	17, 19, 21, 23, 25, 27, 29 = +500 V 18, 20, 22, 24, 26, 28, 30 = −500 V	Δ*x* = −12.5 µm Δ*y* = −3.97 mm Δ*z* = 9.9 µm	All MFCs-F1 type are actuated to generate a clockwise bending moment	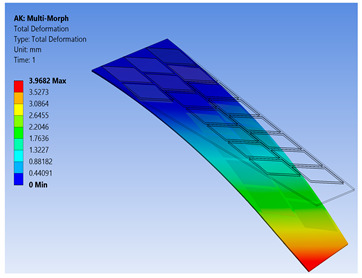
**5**	17, 19, 21, 23, 25, 27, 29 = −500 V 18, 20, 22, 24, 26, 28, 30 = +500 V	Δ*x* = 12.5 µm Δ*y* = 3.97 mm Δ*z* = 9.9 µm	All MFCs-F1 type are actuated to generate a counterclockwise bending moment	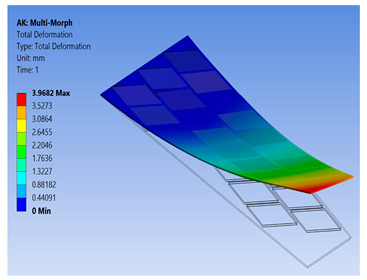
**6**	17 → 30 = −500 V 1 → 16 = +1500 V 31 → 34 = +1500 V	Δ*x* = −0.27 mm Δ*y* = −2.8 mm Δ*z* = 91 µm	Applying configuration 1 with configuration 3	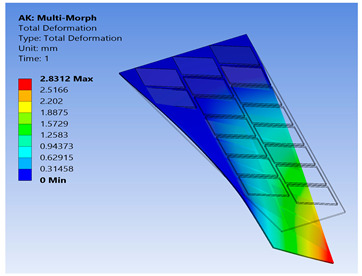

## Data Availability

The data presented in this study are available on request from the corresponding author.
